# Postacute COVID-19 syndrome and fibromyalgia syndrome are associated with anti-satellite glial cell IgG serum autoantibodies but only fibromyalgia syndrome serum-IgG is pronociceptive

**DOI:** 10.1097/j.pain.0000000000003629

**Published:** 2025-05-06

**Authors:** Richard J. Berwick, Peyman Sahbaie, Grace Kenny, Tian-Zhi Guo, Harvey Neiland, David A. Andersson, J. David Clark, Patrick Mallon, Andreas Goebel

**Affiliations:** a Pain Research Institute, University of Liverpool, Liverpool, United Kingdom; b Walton Centre, Longmore Lane, Liverpool, United Kingdom; c Department of Anaesthesiology, Stanford University, Palo Alto, CA, United States; d Centre for Experimental Pathogen Host Research, University College Dublin, Dublin, Ireland; e Department of Infectious Diseases, St Vincent's University Hospital, Elm Park, Dublin, Ireland; f Wolfson Centre for Age-Related Disorders, King's College London, London, United Kingdom

**Keywords:** Post-COVID syndrome, PACS, Long COVID pain, Fibromyalgia, Fatigue, Chronic fatigue, Chronic pain, COVID-19

## Abstract

Postacute COVID-19 syndrome (PACS) describes the persistence of symptoms following severe acute respiratory syndrome coronavirus 2 clearance. PACS is sometimes associated with pain and fatigue resembling fibromyalgia syndrome (FMS). Severe FMS has recently been associated with pronociceptive immunoglobulin G (IgG) autoantibodies and anti-satellite glial cell (SGC) IgG autoreactivity, suggesting an autoimmune aetiology. We validated FMS-IgG passive transfer and then tested the hypothesis that PACS-patients, with high musculoskeletal pain and fatigue, harbour proalgesic and anti-SGC autoantibodies. PACS-patients with high pain and fatigue or people recently recovered from acute COVID-19 were recruited to the All-Ireland Infectious Diseases Study. We pooled serum from 18 patients per group and purified their serum-IgG. In addition, we obtained IgG from UK patients with FMS and healthy controls to confirm assay performance. Passive transfer experiments of IgG (8 mg/d) over 3 days were conducted using male (C57BL/6J) mice (n = 6 mice per group). We measured mechanical and cold hypersensitivities and grip strength. Injection of FMS-IgG elicited the previously described mouse phenotype in male rodents, including increased mechanical/cold hypersensitivities and reduced grip strength compared with control IgG, whereas pooled PACS-IgG was inert. Immunocytochemistry of primary-SGC-enriched cultures reproduced the increased staining of FMS-IgG over the control reported previously. Both IgG from patients with PACS and those recently recovered from COVID-19 stained strongly positive. We confirm the pronociceptive properties of FMS-IgG and demonstrate, in contrast, that PACS symptoms from our cohort, with severe pain and fatigue, are not transmissible through passive transfer to male rodents. Postacute COVID-19 syndrome pain is often localised, and stratification according to the widespread distribution of pain should be considered for future studies; recovered COVID-19 leaves a strong trace of anti-SGC autoreactivity.

## Abstract 2

Supplemental Digital Content is Available in the Text.

Interrogating patients with postacute COVID syndrome with pain and fatigue, we found anti-satellite glial cell IgG autoantibodies that, unlike fibromyalgia syndrome, were not proalgesic in mice.

## 1. Introduction

The recent severe acute respiratory syndrome coronavirus 2 (SARS-CoV-2) pandemic has been the cause of substantial global morbidity and mortality.^19^ Beyond the initial disease, the associated postacute COVID-19 syndrome (PACS, or “long COVID”) has emerged as a source of ongoing health problems.^4,8,40^ PACS is a postinfectious, multisystem disorder, defined as follows: delayed or long-term complications of SARS-CoV-2 infection, existing 4 weeks beyond the onset of symptoms.^40^ The constellation of manifestations is broad and most markedly involves fatigue (often fulfilling criteria for chronic fatigue syndrome), dyspnoea, chest pain, myalgia, arthralgia, headache, cognitive disturbances, dizziness, palpitations (often constituting postural orthostatic tachycardia syndrome): symptoms which all are causing a decline in quality of life.^8,15,28,40,42^ Although PACS is potentially characterised by a persistent inflammatory state, the cause of its symptomatology is largely unknown, though immune and, in particular, autoantibody involvement has been proposed.^1,31,36,45,52^

Recent reports indicate that patients suffering from fibromyalgia syndrome (FMS), a widespread chronic pain condition, that is also associated with significant fatigue, harbour function-modifying immunoglobulin G (IgG) autoantibodies that may explain their symptoms.^25,39^ These patients with FMS had been recruited *before* the COVID-19 pandemic. Upon injection into mice, patient serum-IgG invariably caused significant hypersensitivities in mice, consistent with the patients' clinical symptoms; IgG transfer additionally elicited reduced muscle strength and locomotion, nociceptor hypersensitivity, and skin small fibre neuropathology. Autoantibody reactivity was demonstrated against murine and human satellite glial cells (SGCs), but not to CNS tissues.^25^ Fibromyalgia syndrome pain intensity has additionally been correlated to SGC autoantibody prevalence and titre recently.^20,34^

Although severe fatigue and musculoskeletal pains are also common in PACS, diagnostic criteria for FMS are less frequently met.^50^ Pursuing the possibility of overlapping mechanisms^22,32^ in a subset of patients, we hypothesised that patients reporting severe fatigue and high-intensity musculoskeletal pain, persisting after acute COVID-19 infection, would harbour proalgesic and, potentially, anti-SGC autoantibodies, similar to patients with widespread pain from FMS.

## 2. Materials and methods

### 2.1. Study participants

#### 2.1.1. Non-postacute COVID-19 syndrome participants

For test-validation experiments, serum or plasma from adult (≥18 years) patients, with FMS recruited before the COVID-19 pandemic (except 1) and healthy pain-free, fatigue-free controls (HCs) recruited between 2020 and 2023, was utilised, which was available from an independent phenotyping study^6^ (“APIF,” ISRCTN:18414398). All participants gave written consent, and they were reimbursed expenses up to £30 for their travel and HCs an additional £30 for their time. Waste plasma, retained from clinical plasma exchange treatment from before the COVID-19 pandemic, was additionally utilised from 2 patients who had tested positive for proalgesic-IgG antibodies in our lab, as described previously.^25^ Additional pain-free and fatigue-free HC serum was obtained from the University of Liverpool Pharmacology Biobank (“PHARM B” study) from 2021 to 2023. The patients' demographics and disease characteristics are provided in Supplementary Table 1 (available at http://links.lww.com/PAIN/C280). All sample collections received ethical approval (UK: Ethics North-West Haydock, ref. 15/NW/0467 and 23/NW/0348; or UK: Health and Care Research Board Wales: ref. 18/WA/0234; or University of Liverpool ref: 12267). Samples were collected either at the University of Liverpool premises or at The Walton Centre NHS Foundation Trust, Liverpool, United Kingdom, a neuro-care specialist Hospital, between 2018 and 2023.

#### 2.1.2. Postacute COVID-19 syndrome participants

Patient recruitment was from the All-Ireland Infectious Diseases (AIID) Cohort Study which prospectively recruits individuals with persistent health issues pertaining to any infectious disease. The recruitment details are covered elsewhere^29^ and are outlined here in brief. Adult (≥18 years) subjects, attending Irish infectious disease or long COVID clinics, were routinely invited to participate in the AIID Study. They provided written informed consent for the collection of data on demographics, clinical characteristics, and investigations as undertaken as part of routine care. In this study, we included AIID cohort participants, who earlier had a diagnosis of acute COVID-19 confirmed by positive polymerase chain reaction for SARS-CoV-2, with any symptoms persisting beyond 4 weeks from the date of onset of initial COVID-19 symptoms, as identified in the clinic. No participants were vaccinated before contracting COVID-19. Venepuncture was performed during routine clinical care, usually at the patients' first- but no later than their fifth clinical appointment. At the time of blood donation, patients completed the 36-Item Short-Form Survey (SF-36), as a generic measure of health status and quality of life.^54^ The SF-36 has been shown to be a reliable, valid, and sensitive measure of health status in a variety of clinical settings. Eighteen patients were selected out of 347 patients with PACS who had completed the SF-36 data. We chose patients reporting substantial SF-36 fatigue *and* pain interference at the time of venepuncture. Questions pertaining to energy asked, during the past 4 weeks: (1) “did you feel full of pep,” (2) “did you have a lot of energy,” (3) “did you feel worn out,” and (4) “did you feel tired”? Values from these 4 items (each ranging from 1 to 6) were combined, following inversion for (3) and (4), and converted to a score out of 100 where 0 is maximal fatigue intensity. Questions pertaining to pain asked, “How much bodily pain have you had during the past 4 weeks” and “during the past 4 weeks, how much did pain interfere with your normal work (including both work outside the home and housework)?” The values (each ranging from 1 to 6) from these 2 items were converted to a score out of 100 where 0 is maximal pain impact (a composite of intensity and interference). Eighteen patients who had recovered completely from COVID symptoms (following acute symptoms and a positive COVID serum test but with no specific PACS diagnosis), at 2 to 6 months after the onset of their acute symptoms were also included as a “recovered cohort.” The demographic details are supplied in Supplementary Table 1 (available at http://links.lww.com/PAIN/C280), detailing age, sex, SF-36 pain/energy/fatigue values, and venepuncture timing. In this study, 1.5 mL of serum was supplied per donor. Although the AIID is a pan-Ireland study, our samples were derived only from 2 sites, St Vincent's University Hospital and Mater University Hospital, Dublin. The study was approved in line with National and European regulations on health research by the St Vincent's Hospital Group Research Ethics Committee and the National Research Ethics Committee for COVID-19 in Ireland (ref: RS20-033).

### 2.2. IgG purification and sample pooling

For behavioural and ex vivo experiments, plasma-IgG from plasma exchange-derived waste plasma from 1 patient with FMS and serum-IgG from an age-/sex-matched healthy volunteer from the PHARM B study were purified using large columns as described previously.^25^

As only small volumes were available from the AIID cohort, for the behavioural experiments, 1 pool of all 18 serum samples of PACS and 1 pool of all 18 recovered COVID-19 samples were created using 1 mL of each. These 2 pools were then purified similarly as the large individual FMS or HC samples.

All individual and pooled samples for mouse injection were dialysed against the Hartmann solution, and their concentrations were adjusted to 20 mg/mL.

For the immunohistochemistry experiments, our FMS group included 7 patients in total, 5 patients from the APIF phenotyping study and 2 from plasma exchange, including the one previously used in the behavioural experiments. Of the 7 controls used to compare with FMS, 3 were recruited from the APIF study and 4 from the University of Liverpool PHARM B study.

Postacute COVID-19 syndrome serum samples with high or very high symptom burden from the AIID Study were categorised as such based on their pain and fatigue intensities. This was done by ranking SF-36 fatigue and pain intensity/impact values (out of 100). We selected a “very high” health burden group (G1, n = 6: mean energy 10 [0-25]; mean pain 5 [0-10]) and a “high” health burden group (G2, n = 6: mean energy 16.7 [5-25]; mean pain 11.7 [10-20]). The individual values can be seen in Supplementary Table 1 (available at http://links.lww.com/PAIN/C280). For the recovered cohorts, used as a control for PACS, on which SF-36 data were not available, samples were randomly assigned into 3 groups (n = 6 each) of which in these immunohistochemistry experiments, 1 was tested (RC2). Sera from these 3 groups (G1, G2, RC2) were then pooled equally per group to a total of 500 µL for purification (eg, 83.3 µL per sample).

In addition, we utilised 1 further HC serum sample from the APIF study (not tested as a control for FMS) and 1 of the previously used plasma exchange samples from a patient with FMS (purified by large column IgG purification) as supplementary controls for comparison with the PACS/recovered samples. This HC had been vaccinated against COVID-19 (June 2020, July 2020, September 2021, October 2022) before serum donation (May 2023) and had tested positive for COVID-19 symptoms during the pandemic twice, before donation without the persistence of symptoms. Data on this HC are known due to participation in a COVID-19 vaccine trial.

Purification of these samples was achieved using small HiTrap Protein G HP columns (GE Healthcare), eluted with 0.1 M glycine/HCl pH 2.7, and adjusted to pH 7.4 with 1 M Tris (pH 9).

All concentration measurements, after both large- and small-column IgG purification, were performed using a Nanodrop 1000 (Thermo Fisher Scientific). IgG samples were stored at 4°C for no more than 6 months before testing. In this PACS study, therefore, 2 high burden groups (G1, G2) and 1 recovered group (RC2) were tested for anti-SGC autoantibodies.

### 2.3. Animals

Mice for behavioural studies were 11- to 12-week-old male C57BL/6J strain (000664), obtained from The Jackson Laboratory, Bar Harbor, ME. For SGC staining experiments (including 1 in vitro/in vivo experiment), mice of a C57BL/6J lineage, aged 8 to 10 weeks and weighing 17 to 25 g, were obtained from Charles Rivers UK, Ltd. Female mice were used to be consistent with the literature.^34^ All mice were housed in a temperature-controlled environment with a 12-hour light, 12-hour dark cycle, with access to food and water ad libitum.

### 2.4. Behavioural studies

Mouse behavioural studies were performed at Stanford University, Palo Alto, CA. Before any testing, mice were allowed to acclimatise (10-15 minutes) to the experimental room. Mice were randomised between cages, and the experimenter was blinded to their treatment. To recapitulate the FMS passive transfer experimental approach,^25^ mice were injected on consecutive days (day 1 to day 3) intraperitoneally with 8 mg of test or control IgG (FMS or HC). To assess for the behavioural phenotype: hind paw mechanical sensitivity was measured with paw withdrawal threshold to nylon von Frey filaments according to the “up-down” algorithm.^11^ Thermal sensitivity was measured by a withdrawal latency in the cold plantar assay.^7^ These 2 parameters were tested in groups of n = 6 each for FMS and HC. Forelimb grip strength was measured using a Bioseb GS4 grip strength meter, and mechanical pressure pain threshold was measured with a Bioseb rodent pinch analgesia meter (Bioseb Instruments, Vitrolles, France). These 2 tests were performed in a separate experiment in parallel, n = 6 per group.

Anxiety was assessed with a zero-maze test^46^ in the FMS-IgG injected mice, which also provided an indirect measure of locomotion and general fatigue. This assay was performed at 14 days post first injection, and all FMS-IgG- and HC-IgG-injected mice were included (n = 12 per group).

For the PACS vs recovered-IgG assay, the above was performed with n = 6 per group, but all 4 tests (paw withdrawal threshold, cold plantar withdrawal latency, grip strength, and hind paw pinch) were performed on the same groups because of the sample limited serum supply. An exploratory test of working memory was conducted using a Y-maze test^33^ in the PACS-IgG injected mice to assess for the transmissibility of the cognitive symptoms often reported by patients suffering from PACS, at day 7 post first injection (n = 6 per group).

### 2.5. Cell culture and surface immunocytochemistry

For the in vitro assays, dorsal root ganglia (DRGs) were dissected from mice (46-56 per mouse) according to a protocol adapted from Malin et al.^37^ They were washed with dissociation solution (500 mL Hanks balanced salt solution, 3.5 mL Hepes Buffer, Gilbco) and incubated for 1 hour at 37°C, 5% CO_2_ with 0.3% collagenase (Worthington). After further wash steps, warmed trypsin 0.05% EDTA was added, and DRGs were incubated for 30 minutes at 37°C, 5% CO_2_. The solution was washed and then triturated with a polished glass pipette to form a single-cell suspension. Cells were centrifuged at 160*g* to a pellet, and the dissociation solution was replaced with SGC culture medium (Dulbecco Modified Eagle Medium, 10% B27 [±10% fetal bovine serum (FBS)], 1% penicillin/streptomycin [Sigma]) to select for SGCs.^53^ This solution was filtered twice with a 40-μm cell strainer (Fisher Scientific). For the in vivo-in vitro assay, DRGs were harvested on day 4 from C56/BL6 female mice that had received 3 daily injections of FMS or HC IgG (8 mg).

Two methods for enriched SGC cultures were employed. (A) For the FMS experiments these filtered DRG cells were seeded onto uncoated cover slips to select for glial cell adhesion. Satellite glial cells were allowed to adhere for 90 minutes before media replacement to remove unattached cells (including neurons) and debris. (B) For the PACS samples, the protocol was optimised to enhance cell yield. The dissociated DRG cells were passed through a 10-µm strainer and seeded onto a poly-d-lysine coated coverslip as a concentrated inoculant of 50 µL of 2.5 million cells/mL and allowed to adhere for 5 minutes. Culture media to 1 mL total volume was then added. Cells were incubated overnight at 37°C, with 5% CO_2_ to recover. Coverslips were washed with PBS and subsequently blocked with FBS 10% for 30 minutes and then incubated with 500 µL patient or control IgG in 10% FBS at 10 µg/mL concentration for 3 hours. If staining with anti-glutamine synthetase (Abcam, ab73593 1:500) or anti-major histocompatibility complex class 1 (anti-MHC C1, Invitrogen, ab934635, 1:50) for co-localisation studies in 10% FBS, this was done at the same time. Slides were washed with PBS and fixed with 4% PFA for 10 minutes at 4°C. Secondary antibodies were then added and incubated for 1 hour at room temperature: goat anti-human-Alexa Fluor 488 (Invitrogen, ab2534080, 1:1000 to report human IgG), donkey anti-rabbit-cy5 (Jackson Laboratories, ab2340607, 1:1000 to report glutamine synthetase), or F(ab')2-Donkey anti-Rat IgG (H+L) (Invitrogen, ab2536016, 1:500, to report MHC C1) in 10% normal goat serum (Abcam). Cells were washed in distilled water, and coverslips were mounted with VectaShield hard mount with 4′,6-diamidino-2-phenylindole (DAPI) (VectaShield). Controls for primary and secondary antibody cross-reactivity were performed: primaries without secondaries and the reverse. Antibodies used are shown in Supplementary Figure S1 (available at http://links.lww.com/PAIN/C279).

### 2.6. Image analysis

Satellite glial cell slides were imaged on a Zeiss LSM780 confocal microscope with LSM ZEN2012 (Zeiss) software at the Centre for Cell Imaging, University of Liverpool (equipment supported by an MRC grant MR/K015931/1). The background signal was determined by a negative primary control. For imaging of dissociated DRG cells, z-stacks of 1 to 3 images were taken with a pinhole of 1 airy unit of 3 to 5 regions. These images were combined through a maximum intensity projection, with image resolution optimised for settings and focal plane. The process was optimised using automated tile scanning to enhance total cell numbers from the PACS samples. Images were processed with Fiji (ImageJ v2.0.1). An open access machine learning algorithm, “Cellpose,” was used to identify cells by defining a region of interest using default settings and the “Cytoplasm2” model.^49^ Segmentations were performed in the transmitted light channel and quality-controlled by hand. Regions of interest were confirmed as cellular with DAPI signal and pixel data tabulated, including mean, median, mode, maximum, minimum, integrated pixel intensity density, and area, using Fiji. Cells over 120 µm^2^ and of non-SGC morphology were excluded as unlikely to be of SGC origin given the chosen culture conditions. Soma size has been found to be a reliable determinant of nonneuronal cell identity (ie, of SGCs), with a 15-µm diameter serving as a clear cut-off.^47^ We used brightfield morphology (ovoid, <12 µm diameter, with minimal cytoplasm), high DAPI nuclear staining, and cell area (<120 µm^2^) to identify SGCs for IgG staining measurement. We, additionally, used glutamine synthetase reactivity for imaging co-localisation.

### 2.7. Power calculations

Power calculations for the behavioural studies suggested that n = 6 mice per group would suffice to detect a 25% reduction in the paw withdrawal threshold with α < 0.05 and 1 − β > 0.8 (6 repeated measures, 2-sided), which is sufficient to detect a reduction in the cold-plate paw withdrawal latency reduction of 15%.^25^

### 2.8. Statistical analysis

For behavioural studies, we used repeated measure analysis of variance followed by either Šidák or Dunnett post hoc test. Differences in the immunofluorescence signal intensities of SGC staining were analysed by a Mann-Whitney test or a Kruskal-Wallis test with a Dunn post hoc evaluation. When analysing total cell immunofluorescence data, median, intensity density, and maximum pixel intensities are shown. In the scatter figures, medians and interquartile ranges are displayed as the data are nonnormally distributed. Cell intensity density was calculated; however, as this is a measure of the total amount of signal from a whole cell, it is possibly confounded by cell size. Because cell segmentation inaccuracies may lead to variation in pixel intensity data, especially, due to the small cell size (often <10 µm diameter), maximum pixel intensity was selected as a more consistent measure and used to confirm the differences seen in the other variables. This approach also demonstrated whether there was any ceiling effect caused by overexposure. In the figures pixel, median, intensity density, and maximum intensity values are displayed. No data have been removed.

## 3. Results

**Figure 1.**
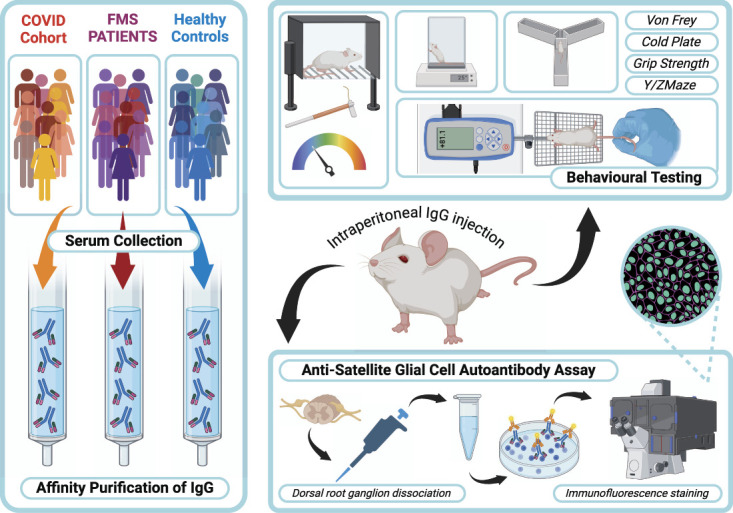
Graphical representation of study protocol. Created in BioRender. https://BioRender.com/h64x676.

The clinical demographics of patient cohorts are displayed in Table 1. The FMS cohort and PACS cohorts G1 and G2 were not statistically different from each other although G3 was a less severe phenotype. The experimental protocol is summarised in a graphical diagram (Fig. 1).

**Table 1:** Baseline characteristics.

	FMS	PACS G1^a^	PACS G2^a^	PACS G3	R1	R2^a^	R3	HC
n	7	6	6	6	6	6	6	8
Sex (M:F)	0:7	1:5	0:6	1:5	1:5	1:5	1:5	1:7
Age (y)	57.0 (38.0-61.0)	40.5 (38.5-51.3)	41.5 (37-51.3)	60.0 (56.8-74.5)	49.5 (34.0-70.3)	48.0 (38.0-72.0)	60.0 (37.5 75.8)	40.0 (31.3-53.5)
Energy impact	10.0 (9.0-10.0)^b^	7.5 (0.0-21.3)	20.0 (8.8-21.3)	42.5 (35.0-46.3)	—	—	—	—
Pain impact	9.0 (7.0-10.0)^b^	5.0 (0.0-10.0)	10.0 (10.0-12.5)	22.5 (22.5-22.5)	—	—	—	—
Blood draw (d)	—	172 (97.5-221.3)	180.5 (91.3-230.8)	164 (64.8-261.5)	77.5 (57.0-148.5)	71.0 (50.8-129.3)	51.0 (47.8-135.8)	—
Vaccinated^c^ (n)	0/7	2/6	0/6	4/6	0/6	0/6	0/6	1/8

Blood draw following symptom onset. ^a^PACS samples tested in staining assays. ^b^For FMS, the pain and fatigue scores were taken directly from NRS scores and are not included in the statistical calculations but are illustrative only (10 is maximum, 0 is minimum). ^c^All vaccination occurred before infection. Median and interquartile ranges are shown. Pain and energy impact for the AIID groups are shown as values out of 100, where 0 is maximal interference and 100 is no interference. Statistical significance is shown with the Kruskal-Wallis test with Dunn post hoc analysis applied. For energy, G1 < G3** and G2 < G3* where a lower score is more severe. For pain interference G1 < G3*** and G2 < G3*—again where a lower score is more severe. Age and blood draw were not significantly different between groups. *P < 0.05, **P < 0.01, ***P < 0.01. — = data not available.

AIID, All-Ireland Infectious Diseases; FMS, fibromyalgia syndrome; NRS, Numeric Rating Scale; PACS, postacute COVID-19 syndrome.

### 3.1. Fibromyalgia syndrome serum-IgG from a UK patient donor causes hypersensitivity in the passive transfer model but pooled serum-IgG from patients with postacute COVID-19 syndrome does not

First, we sought to confirm the performance of our FMS passive transfer model with behavioural tests employed at the collaborating laboratory, using plasma/serum-IgG from 1 UK patient with FMS who was previously tested positive at a different laboratory,^25^ and 1 HC. We then investigated pooled serum-IgG from 18 individuals with PACS from the AIID cohort vs 18 recovered individuals in the same model. The average pain score of the FMS individual was likely less severe (6/10; 10 = maximal) than the PACS cohort where the impact score was (13/100 overall, where 0 = maximal).

**Figure 2.**
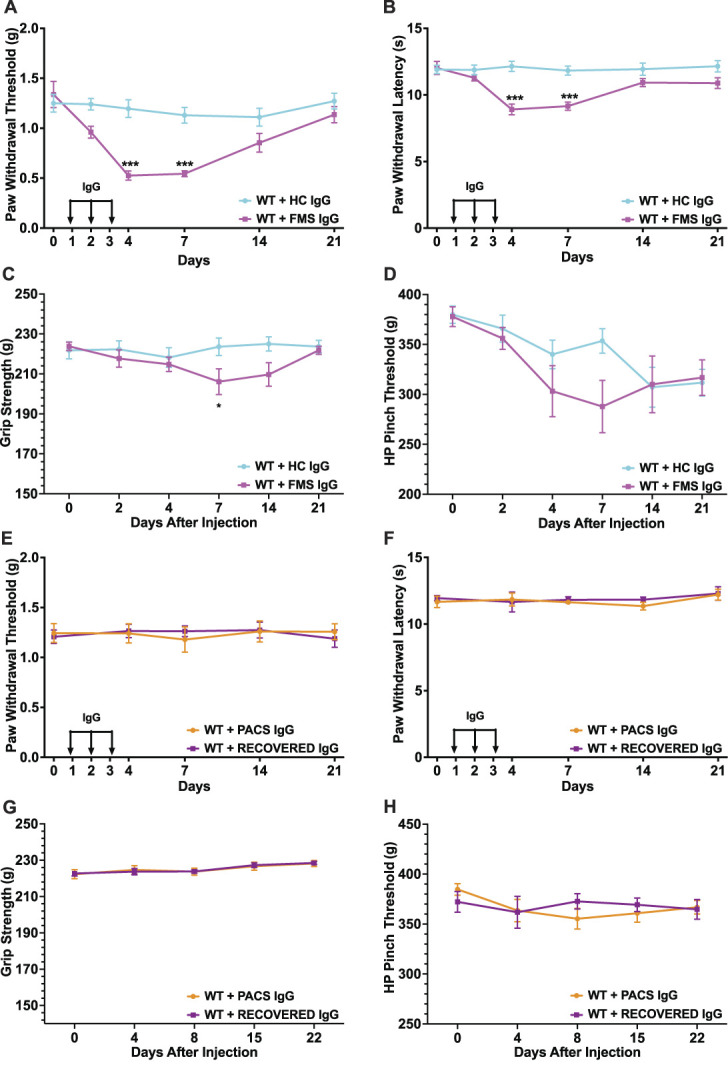
Behavioural tests of FMS/HC and PACS/recovered serum-IgG preparations. (A) Hind paw withdrawal thresholds, (B) cold sensitivity measurements, (C) grip strength, and (D) hind paw pinch hyperalgesia measurements of C57Bl/6J male naive mice after 3 days of systemic IgG injection from 1 FMS patient plasma and 1 healthy control serum, n = 6 mice per group for (A + B) and n = 6 for (C + D). (E) Hind paw withdrawal thresholds, (F) cold sensitivity, (G) grip strength, and (H) pinch hyperalgesia in C57Bl/6J male naive mice after 3 days of systemic IgG injections from PACS and recovered patient sera, n = 6 mice per group. The FMS sample is noted as F101 and the HC, H101, details in Supplementary Table 1 (available at http://links.lww.com/PAIN/C280). A total of 18 mice were used. Mean and SEM shown. Significance tested with 2-way analysis of variance with Šídák multiple comparisons test. FMS, fibromyalgia syndrome; HC, healthy control; PACS, postacute COVID syndrome; RECOVERED, recovered acute COVID infection; WT, wild-type mice. ***P < 0.001, *P < 0.05.

Fibromyalgia syndrome-IgG demonstrated the previously reported, typical patient phenotype compared with controls (Figs. 2A–D). By day 4, the FMS-IgG injections had caused a significant reduction in the mouse paw withdrawal threshold (mechanical hyperalgesia) and latency (cold hyperalgesia) compared with HC-IgG, which normalised after 14 days (Figs. 2A and B); grip strength was reduced at day 7 (Fig. 2C). No difference was seen in hypersensitivity to pinch threshold (previously not tested, Fig. 2D). A zero-maze test of anxiety was conducted at 14 days showing no difference between FMS-IgG and HC-IgG injected male mice (previously not tested) in total travel or open arm distance (seen in Supplementary Fig. S2, available at http://links.lww.com/PAIN/C279).

Pooled PACS-IgG from those with pain failed to generate significant abnormalities in any of the behavioural tests (Figs. 2E–H). Paw withdrawal thresholds, cold withdrawal latencies, grip strength, and hypersensitivity to pinch were indistinguishable between PACS and control groups. Working memory evaluation was also measured with a Y-maze test, as a proxy of the cognitive symptoms seen in PACS (Supplementary Fig. S2, available at http://links.lww.com/PAIN/C279). This measure showed no difference compared with control IgG-injected mice.

### 3.2. Fibromyalgia syndrome serum-IgG from UK patients contains anti-satellite glial cell antibodies

**Figure 3.**
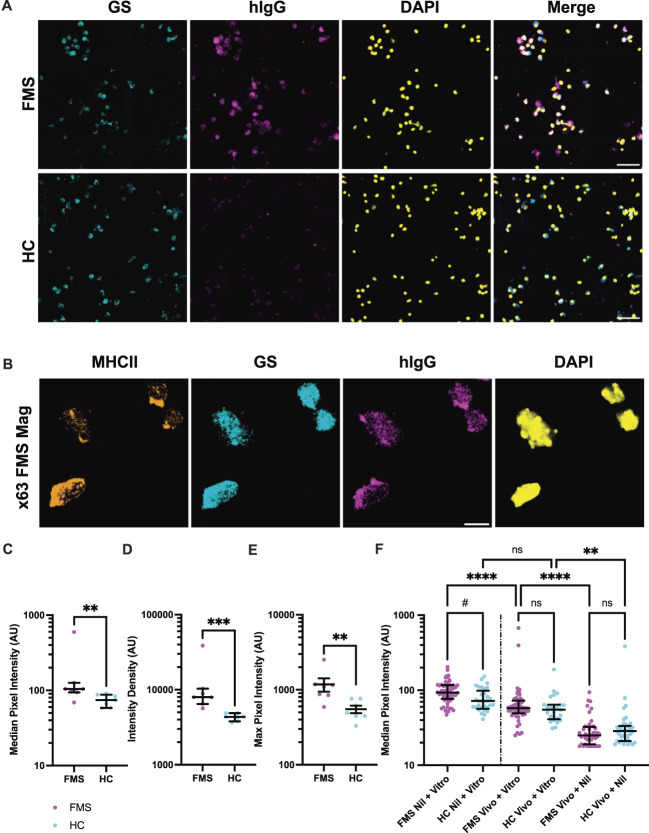
Immunofluorescence staining of satellite glial cells. (A) Typical appearance of immunofluorescence staining of SGC live-cell cultures following incubation with either FMS or HC. In B-E, the median, intensity density, and maximum pixel intensities for all pixels of each SGC stained with IgG of the 7 individual FMS/HC sample-pairs were calculated. The median of each of these IgG-preparation pairs was taken and is shown in the plots with the median and interquartile range overlaid. (B) Median of mean, (C) median of median, and (D) median of maximum, pixel intensity data for all SGCs stained with IgG of 7 individual FMS/HC sample-pairs. (E) Median pixel intensity of cells from primary enriched SGC cultures stained with FMS or HC IgG either in vitro only, in vivo and in vitro combination, or in vitro only. Here, each dot represents the data from 1 cell. The data from “FMS Nil + Vitro” and “HC Nil + Vitro” come from a separate experiment which contributed to the data displayed in B-D. The FMS and HC preparations used in E are the same as those used in the behavioural experiment, Figure 1A–F; a different preparation from the same patient and HC was independently small-column purified and comprised 1 of the 7 pairs in B-D. DAPI, 4′,6-diamidino-2-phenylindole; FMS, fibromyalgia syndrome; GS, glutamine synthetase; HC, healthy control; SGC, satellite glial cell. Scale bar is 50 µm. ****P < 0.0001, ***P < 0.001, **P < 0.01, #*P* < 0.001 when analysed as separate experiment (Mann-Whitney *U* test) from the day performed, though *P* < 0.097 when combined in total analysis (Kruskal-Wallis, with Dunn post hoc adjustment).

To cross-validate published data about SGC serum-antibodies from Swedish and Canadian cohorts obtained at a different laboratory,^25,34^ we next sought to confirm the presence of anti-SGC antibodies in the sera/plasma of UK patients with FMS (Fig. 3A).

Live-cell immune staining of SGC-enriched cultures was performed ensuring that predominantly cell surface antigens were exposed for IgG binding, during incubation with individual FMS or HC serum-purified IgG. Human IgG staining, as detected by a secondary reporter antibody, seemed to be predominantly on the SGC surface, although, given the diminutive size of the cells, it only occasionally demonstrated a ring pattern (ie, when the focal plane coincided with the cell mid-section). This is most evident at higher magnification (×63) in Figure 3B, where staining for the MHC C1, a cell surface marker, was performed to assess co-localisation. The human IgG staining pattern aligns more closely with MHC C1 than with the intracellular marker glutamine synthetase, suggesting its association with cell surface antigens. The amount of patient, or control, IgG binding was quantified by cell immunofluorescence using the secondary antibody raised to human IgG (Figs. 3C–E). The median, intensity density, and maximum pixel intensities were seen to be significantly increased with FMS-IgG staining overall (Figs. 3A–D) and were also significantly stronger than for HCs in each of the 7 experiments, in at least 1 parameter of mean, median, maximum, or intensity density values (Supplementary Fig. S3, available at http://links.lww.com/PAIN/C279). In some samples, intense speckling was noted, and, in these samples especially, maximum intensity data better captured the differences between FMS and HC samples. Maximum intensity also gave a sense of intensity saturation.

We theorised that antibody-epitope interactions might influence epitope availability, possibly through upregulation by signal transduction mechanisms, epitope stabilization, or trafficking interference. We conducted FMS passive transfer using a preparation of FMS-IgG known to be (1) active in previous passive transfer experiments^25^ and (2) associated with in vitro SGC staining (Fig. 3F). We then incubated SGC cells derived ex vivo from these mice, in vitro with the same FMS or HC-IgG (“FMS Vivo + Vitro”) followed by a secondary reporter antibody and compared these results with the incubation of the same FMS or HC-IgG in vivo only, followed by the secondary reporter antibody, that is, without in vitro primary incubation (“FMS Vivo + Nil”). This was compared with SGC staining from the same samples in vitro only, from another experiment that had shown the expected FMS > HC staining pattern. In the in vivo-only condition, immunofluorescence was low for both FMS and HC, which we suspect was due to IgG being stripped from surface epitopes during dissociation. Unexpectedly, however, median immunofluorescence data (Fig. 3F) showed that mice injected with IgG before DRG harvest, and subsequently incubated in vitro with IgG, exhibited reduced binding in both groups compared with the in vitro-only incubation. This reduction was more marked in the FMS group, diminishing the significant difference (FMS > HC) seen with in vitro-only incubation.

### 3.3. Postacute COVID-19 syndrome patient sera and recently COVID recovered sera contain anti-satellite glial cell antibodies

**Figure 4.**
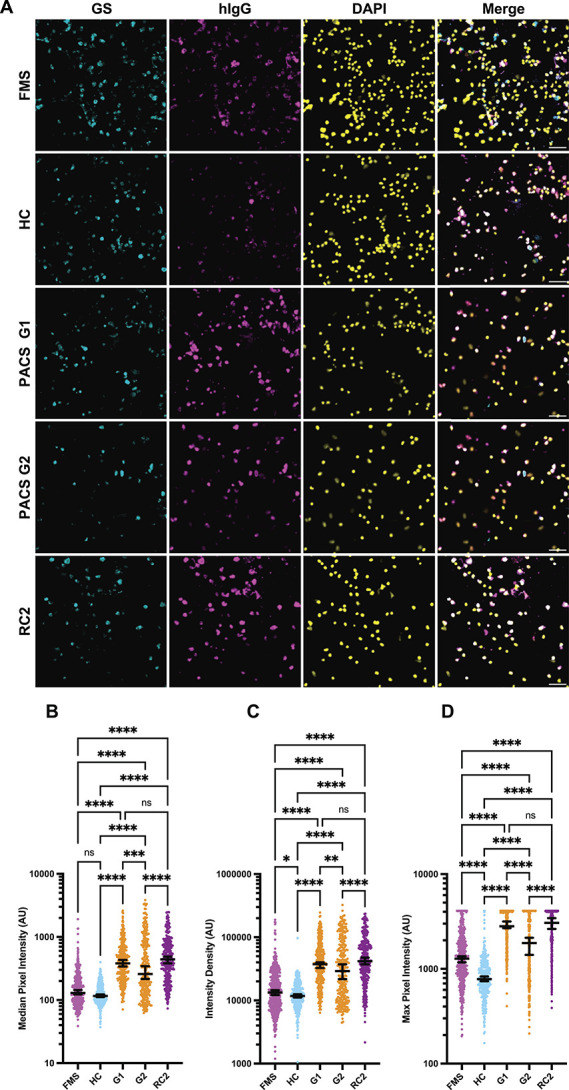
Immunofluorescence staining of satellite glial cells. (A) Typical appearance of immunofluorescence staining of SGC live-cell cultures following incubation with either FMS, HC, PACS G1, G2, or RC. (B-D) Median, intensity density, and maximum intensity immunofluorescence staining data of 1 primary-SGC-enriched culture with IgG from a subject with FMS, a healthy control patient, 2 pooled painful PACS cohorts, G1 and G2 (n = 6 each), and a recovered, RC2 cohort (n = 6), taken from the cohorts used in the passive transfer model. Median and interquartile range are shown in each. Each dot represents the data from 1 cell. Cells included in the analysis were HC = 333 FMS = 449, RC2 = 283, G2 = 272, G1 = 302. Significance tested with Kruskal-Wallis, with Dunn post hoc adjustment. Scale bar is 50 µm. FMS, fibromyalgia syndrome; HC, healthy control; G1, group 1 PACS; G2, group 2 PACS; PACS, postacute COVID-19 syndrome; RC2, recovered COVID-19; SGC, satellite glial cell. ****P < 0.0001, **P < 0.01, ns = not significant.

Next, we performed the live SGC cell staining experiments with the PACS cohort (Figs. 4A–D, Supplementary Fig. S4, available at http://links.lww.com/PAIN/C279). We compared a “very high” PACS symptom group (G1 [n = 6, fatigue: <25, pain: ≤10 see methods]) and a “high” symptom group (G2 [n = 6, fatigue: 5-25, pain: 10-22.5]) against a recovered acute COVID-19 (RC2) group (n = 6). For a negative control, we used an HC, and for a positive control, we used a patient with FMS (pain 25/100) with known pronociceptive and SCG staining antibodies (which was also used in the SCG staining experiments—see Supplementary Table 1, available at http://links.lww.com/PAIN/C280).

Staining of both pooled PACS groups and the pooled recovered group was very high, indistinguishable from each other, and much stronger than the FMS positive control.

Mean intensity showed the expected FMS > HC staining pattern, although the median FMS vs HC intensity was just outside the 0.05 cut-off (0.0509, Dunn post hoc test). Maximum values showed that there was a ceiling effect or saturation for FMS, G1, G2, and RC2. The control patient, although, technically, a patient uniquely recovered from COVID-19 (as they received vaccination before infection), stained SGC cultures with similar intensity as other HCs, and all were less than the PACS groups (Figs. 2B–D and Supplementary Fig. S5, available at http://links.lww.com/PAIN/C279).

## 4. Discussion

This study confirms successful IgG-passive transfer of FMS symptoms, including hyperalgesia and reduced muscular power in male mice, replicating previous findings.^25^ Hypothesising that painful PACS may be sustained by IgG-autoimmune mechanisms, similar to FMS, we found, however, no proalgesic effects of IgG from patients suffering from PACS with high-intensity pain and fatigue (“PACS-pain”).

In FMS-IgG passive transfer, rodent behavioural readouts mirror the patient phenotype of widespread mechanical hypersensitivity. Clinically, by contrast, PACS exhibits a more nuanced and heterogenous sensory profile, often featuring localised paraesthesias rather than widespread mechanical sensitivity.^41,43^ Interestingly, 34% of patients with PACS report neuropathic symptoms, while 40% to 50% of patients with FMS display small fibre abnormalities.^26^ Sensory phenotyping may enable stratification of patients by small fibre pathology, revealing potential differences in subgroup pathophysiology.^21^ Neuropathic pain, seen in Guillian Barré syndrome^55^ and small fibre neuropathies,^17^ can be autoantibody-driven, possibly through interactions with neuronal channels (eg, potassium^30^) FcγRs, neuroinflammation from glial/Schwann cell damage,^35^ or, maybe, endothelial dysfunction, implicated in PACS.^18^

Our in vitro experiments replicated findings from Canadian and Swedish FMS cohorts of enhanced FMS-IgG SGC staining over controls, in UK patients. We also observed that in vivo exposure to FMS- or HC-IgG reduces surface binding structure availability on SGCs. The FMS-IgG induced *reduction* (Fig. 3F, In Vitro_Nil cf. In Vivo_In Vitro) appeared stronger compared with the HC-IgG reduction, diminishing the in vitro staining differences between FMS and HC samples. This suggests that a dynamic process shaping surface structures on SGCs in vivo has led to reduced expression following FMS-IgG exposure. These findings might reflect a process by which FMS-IgG autoantibody clustering induces epitope internalisation and lysosomal degradation, potentially altering cell function.^23^ Whether this process is relevant for the transferred FMS phenotype requires further study. The alternative explanation for low in vivo-in vitro staining—that in vivo IgG further blocks in vitro binding through epitope masking—appears unlikely, since, following enzymatic DRG dissociation, almost no reporter-recognisable IgG was detected in the in vivo samples available to block in vitro binding.

Furthermore, we found that both patients with PACS (<1-year duration) and, unexpectedly, individuals recently fully recovered from COVID-19 exhibit high anti-SCG IgG antibody titres. Pain levels in high pain/fatigue intensity groups G1 and G2 exceeded those observed from the patient with FMS (Table 1 and Supplementary Table 1, available at http://links.lww.com/PAIN/C280). Satellite glial cell staining intensity correlates with pain scores.^34^ However, as RC2 (recovered) participants, reporting no pain or fatigue, demonstrated equally strong staining, anti-SGC staining titre *alone* seems *insufficient* to explain symptoms in PACS. Instead, high staining in the recovered PACS group likely indicates a persistent immunological imprint from recent infection (2-6 months prior). In early PACS samples, collected 6 to 12 months postinfection, PACS-pain-related effects are indistinguishable from the acute COVID-19 infection imprint. Notably, 1 UK control sample, recruited later in the pandemic and extensively vaccinated, showed low anti-SGC antibody titres despite a prior COVID-19 infection. This suggests vaccination reduces off-target antibodies in the B-Cell response, consistent with existing research on post-COVID-19 IgG autoreactivity.^51^ It also indicates that an anti-SGC serum test is unsuitable for diagnosing PACS pain, up to 1 year after infection. Autoimmune dysregulation is common in acute COVID-19, with high titres of antibodies to connective tissue, vascular endothelium, and CNS tissue,^52^ including autoantibodies (ACE2,^3^ β2-adrenoceptors, and muscarinic M2 receptors^51^). Subsequently, if PACS developes, immune profiles reveal elevated circulating activated B cells,^31^ exhausted T cells, and immune dysregulation, certainly conducive to abberant antibody selection.

Importantly, the target of anti-SGC antibodies in FMS remains unknown. The binding of anti-SGC IgG in FMS, with titres lower than in PACS, might indicate different molecular targets. Despite all patients with FMS having symptoms for over 2 years, unlike the relatively recent onset in patients with PACS, research suggests that SGC binding intensity is unlikely explained by disease duration.^34^ Future studies might examine the importance of serum anti-SGC antibodies by selective depletion or enrichment from patient IgG, before murine passive transfer.

Our hypothesis that PACS symptoms can be transferred through IgG was not upheld in this cohort. Superficially, this contrasts with Chen et al.'s recent pre-pub, which suggests that IgG from patients with PACS—with high neuronal or leucocyte activation proteins titres but not skeletal muscle proteins—are proalgesic upon passive transfer.^12^ The absence of symptom transfer in our cohort indicates that the pooled PACS-IgG was not pathologically autoreactive (similar to Chen et al.'s negative skeletal muscle protein group) or that an unknown adjuvant, as in CRPS,^24^ is required.

Defining the pain phenotype in patients with PACS is crucial, as PACS pain is likely heterogeneous. Musculoskeletal pain from overuse (eg, coughing) or underuse (eg, deconditioning) may mimic any autoimmune somatosensory pain and elevate serum muscle protein breakdown products but is unlikely to transfer to mice. The anatomical distribution of pain in our patients with PACS was unknown, and despite having *severe* pain impact, they may not have experienced the “widespread” pain characteristic of FMS.^22,32,50^ If autoimmune factors contribute to severe *regional* PACS pain, IgG transfer may not induce the ubiquitous rodent sensitivity required for behavioural readouts. Notwithstanding, other mechanisms, such as central sensitisation,^13,14,48^ may underlie widespread PACS pain. Further studies with precisely phenotyped patients with PACS are needed to correlate clinical presentation with pathophysiology, as pain/fatigue-based phenotyping alone is insufficient to clarify the role of *systemically-acting* proalgesic-IgG autoantibodies in PACS.

Interestingly, our passive transfer results in a single patient with FMS, in this study, while consistent with the first FMS passive transfer report, contrast with a subsequent study reporting no effect. The latter used a single 8-mg IgG injection from a patient with an uncharacterised phenotype.^9^ Given that IgG-autoantibody titers in FMS may correlate with pain intensity^20,34^ and rodent proalgesic behaviour depends on injected IgG mass,^16,25^ patient selection is critical; phenotype expression reflects both pain intensity and IgG titre. In addition, IgG purification methods differ, and the negative study^9^ used protein A, which poorly extracts IgG3.

We previously observed a depressive effect on nocturnal rodent locomotion following IgG transfer from UK patients with severe FMS, potentially consistent with fatigue symptoms.^25^ The patients with PACS, in this study, exhibited low SF-36 energy scores indicating strong fatigue. However, we observed no ambulatory abnormalities in PACS-IgG treated mice, either in their cages or in the Z-maze test, which—though primarily assesses anxiety—involves voluntary movement.

Cognitive symptoms are frequently associated with PACS.^40^ We found no difference in the spatial working memory of mice injected with PACS-IgG. This is only 1 aspect of cognitive function, again highlighting the importance of assessing biological factors in patients who are well-phenotyped, permitting subgroup clustering.

Our study had several limitations. Testing mechanistic similarities between PACS pain and FMS was limited by incomplete PACS cohort phenotyping. Despite high musculoskeletal pain, patients were not assessed against the current FMS diagnostic criteria.^5^ Similarly, Chen et al.'s study lacks pain severity and location data, complicating interpretation.^12^ Our PACS-pain cohort was sampled at a mean of 6 months post-symptom onset (2-12 months), compared with 3 months (3-6 months) in the recovered cohort—a potential confounder, as postinfectious IgG titres change over time.^2^

For immunostaining, live staining targeted cell surface antigens, but, active IgG cell uptake during incubation, may shielded it from the secondary antibody reporter. This could be tested with a pH-sensitive reporter.^44^ Some saturation was observed during imaging, though exposure was standardised for experimental comparability.

We pooled PACS serum samples to ensure sufficient material for passive transfer studies, assuming additive or synergistic reactivities among contributing sera. However, pooling may dilute proalgesic-IgG antibodies from some individuals or skew staining results due to extremely high autoantibody titres. However, this method has been successfully used before in passive transfer experiments, faithfully reproducing the results from individual samples.^25^ Pooled PACS-IgG from G1-G3 stained SGCs strongly, and participants had *stronger* median pain impact compared with the FMS control (Supplementary Table 1, available at http://links.lww.com/PAIN/C280).

Our study employed male mice for behavioural assessments and female mice for in vitro staining, following Goebel et al.,^25^ which reported no significant sex-dependent differences in IgG-mediated hypersensitivity. This approach aimed to minimise hormonal variability and reduce animal numbers. Notably, Chen et al.^12^ reported no sex differences in IgG-mediated PACS. However, recent literature indicates that pain mechanisms may involve sex-specific pathways not fully represented in male-only studies.^38^ Consequently, future experiments incorporating female rodents are warranted to determine whether PACS-IgG-mediated effects differ between sexes. We focused on downstream autoantibody mechanisms, but sex-specific pathways could influence antibody production in PACS, as emerging evidence suggests sex-specific host immune responses.^27^ In addition, while our staining experiments in female mice ensured consistency with prior SGC studies,^34^ there is a need to directly compare immune and pain responses across sexes.

Pain and fatigue are canonical features of both FMS^5,50^ and PACS. The presence of autoantibodies in PACS may reflect impaired self-tolerance,^10,12^ a mechanism potentially shared with FMS.^39^ In this study, while we detected a high titre of anti-SGC IgG antibodies in the sera of a patient with PACS, cellular reactivity was not associated with a mouse pain-sensitising effect. It appears that COVID-19 infection is associated with SGC autoreactivity, irrespective of pain symptoms. Given the possibility of antibody-mediated PACS symptoms^12^ and the apparent prevalence of specific PACS autoantibodies,^10^ further investigation is warranted, with detailed phenotyping of patients, including stratification for pain “widespreadness”.^39^

## Conflict of interest statement

A. Goebel receives consultancy fees from UCB. R. J. Berwick receives consultancy fees from BioHaven and previously UCB. The other authors have no conflicting interests to declare.

## Supplemental digital content

Supplemental digital content associated with this article can be found online at http://links.lww.com/PAIN/C279 and http://links.lww.com/PAIN/C280.

## Supplementary Material

SUPPLEMENTARY MATERIAL
