# Supplementary material for: Postacute COVID-19 syndrome and fibromyalgia syndrome are associated with anti-satellite glial cell IgG serum autoantibodies but only fibromyalgia syndrome serum-IgG is pronociceptive
Source: Pain. 2025 May 6;166(10):e397–408. doi: 10.1097/j.pain.0000000000003629 (PMC12444900; doi:10.1097/j.pain.0000000000003629)
Supplement: SUPPLEMENTARY MATERIAL [file jop-166-e397-s002.pdf]

Figure S1

Antibodies used:

Antibody	Manufacturer	Host	Concentration	RRID
**Primaries**				
Human IgG	*Protein G purified*	*NA*	*10μg/ml*	*NA*
Anti-Glutamine Synthetase	*Abcam*	*Rabbit*	*1:500*	*AB_73593*
MHC I Polymorphic Monoclonal Antibody (ER-HR52)	*Invitrogen*	*Rat*	*1:50*	*Ab_2536016*
Secondaries				
Anti-Human-Alexa Fluor 488	*Invitrogen*	*Goat*	*1/1000*	*AB_2534080*
Anti-Rabbit CY5	*Jackson Laboratory*	*Donkey*	*1/1000*	*AB_2340607*
F(ab')2-Donkey anti-Rat IgG (H+L) Highly Cross-Adsorbed Secondary Antibody, TRITC	*Invitrogen*	*Donkey*	*1:500*	*Ab_2536016*
**Blocking Serum**				
Normal Goat Serum	*Abcam*	*Goat*	*10%*	*AB_7481*
Fetal Bovine Serum	*Invitrogen*	*Cow*	*10%*	*NA*

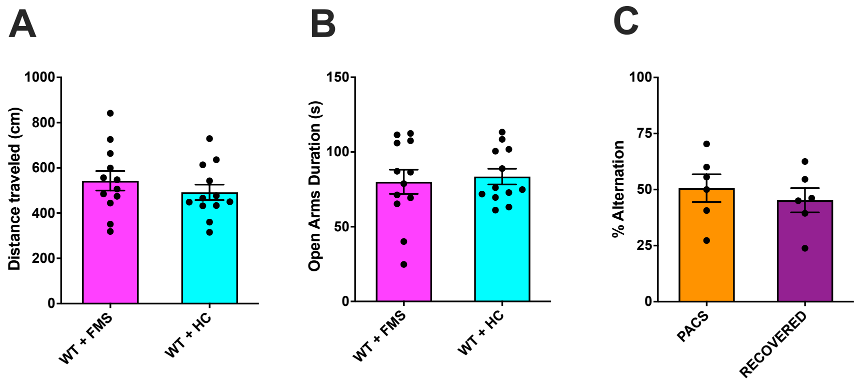

Figure S2 - *Additional Behavioural tests of FMS/HC and PACS/recovered serum-IgG preparations.* (A) Open arm duration and (B) distance travelled in a zero-maze test of anxiety at day 14 in the animals injected with FMS-IgG, n=12 mice/group. (C) Spatial working memory evaluations were made using the Y-Maze in mice injected with PACS-IgG or recovered-IgG, 7 days after initiation of treatment with IgG from both groups (n=6 mice/group). Mean and standard error of means shown. Abbreviations: FMS=fibromyalgia syndrome; HC=healthy control; PACS=post-acute COVID syndrome; RECOVERED=recovered acute COVID infection; WT=wild type mice. Statistics - Mann Whitney U Test. No significant difference found.

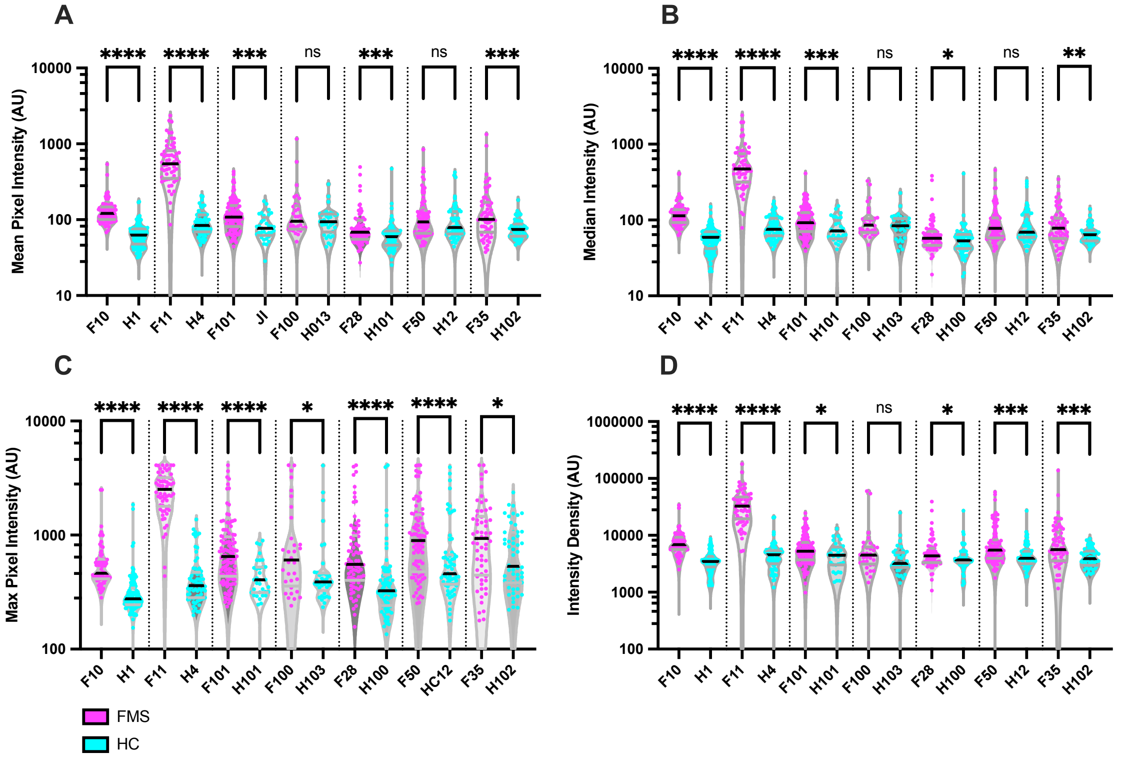

Figure S3 - *Complete immunofluorescence staining data of satellite glial cells for FMS vs HC assay.* (A) Mean, (B) Median, (C) Max pixel intensity and (D) Intensity density data for all SGCs stained with IgG of seven individual FMS/HC sample-paired experiments. Median and interquartile ranges are marked on the violin plots. (E) Intensity density plot showing median of intensity density of all SGC data for each FMS vs HC pair. FMS is in magenta and HC in cyan. Abbreviations: FMS=fibromyalgia syndrome; HC=healthy control. Statistics - Kruskal-Wallis with Dunn's Post Hoc test. $p<0.0001$ ****, $p<0.001$ ***, $p<0.01$ **, $p<0.01$ *, ns=not significant.

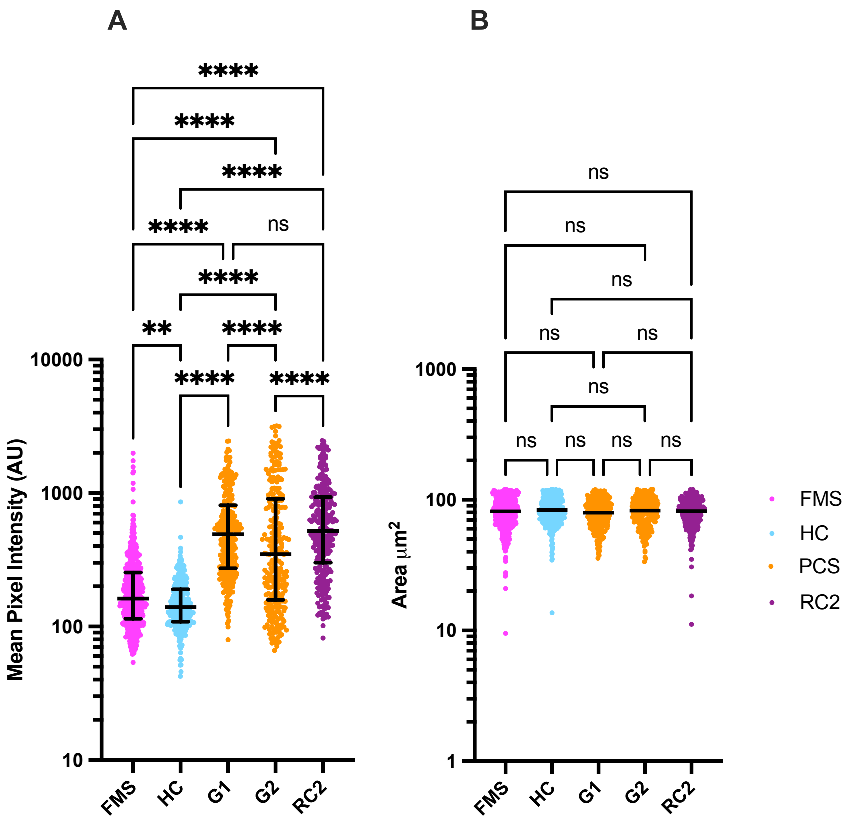

Figure S4 - *PACS immunofluorescence staining data of primary SGC enriched cultures*. (A) mean intensity values and (B) cell area of primary cell culture stained with IgG from a subject with FMS, a healthy control patient, two pooled painful PACS cohorts, G1 and G2 (n=6 each) and a recovered, RC2 group (n=6) taken from the cohorts used in the passive transfer model. Median and interquartile range are shown in each. Abbreviations: FMS=fibromyalgia syndrome; HC=healthy control; G1=Group 1 PACS; G2=Group 2 PACS; RC=recovered COVID. Statistics - Kruskal-Wallis with Dunn's Post Hoc test. p<0.0001 ****, p<0.01 **, ns=not

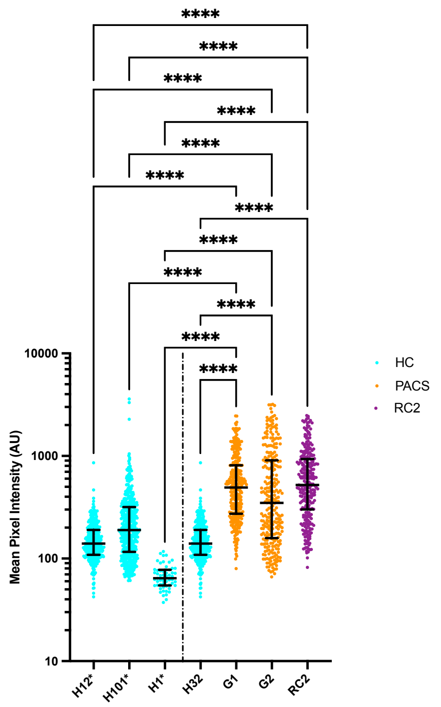

Figure S5 - *PACS immunofluorescence staining data of primary SGC enriched cultures* compared to data from other healthy controls. Mean intensity signal of cells from SGC enriched cultures stained with IgG from the experiment in S3 with additional controls (*) from other similar experiments using the same SGC isolation technique Abbreviations: Healthy controls (H12, H101, H1, H32); FMS=fibromyalgia syndrome; G1=Group 1 PACS; G2=Group 2 PACS; RC2=recovered COVID. Statistics - Kruskal-Wallis with Dunn's Post Hoc test. $p<0.0001$ ****
